# Multivalent Human Papillomavirus L1 DNA Vaccination Utilizing Electroporation

**DOI:** 10.1371/journal.pone.0060507

**Published:** 2013-03-25

**Authors:** Kihyuck Kwak, Rosie Jiang, Subhashini Jagu, Joshua W. Wang, Chenguang Wang, Neil D. Christensen, Richard B. S. Roden

**Affiliations:** 1 Department of Pathology, Johns Hopkins University, Baltimore, Maryland, United States of America; 2 Department of Biostatistics, Johns Hopkins University, Baltimore, Maryland, United States of America; 3 Departments of Pathology, Microbiology and Immunology, Penn State University, Hershey, Pennsylvania, United States of America; National Institute of Health - National Cancer Institute, United States of America

## Abstract

**Objectives:**

Naked DNA vaccines can be manufactured simply and are stable at ambient temperature, but require improved delivery technologies to boost immunogenicity. Here we explore in vivo electroporation for multivalent codon-optimized human papillomavirus (HPV) L1 and L2 DNA vaccination.

**Methods:**

Balb/c mice were vaccinated three times at two week intervals with a fusion protein comprising L2 residues ∼11−88 of 8 different HPV types (11−88×8) or its DNA expression vector, DNA constructs expressing L1 only or L1+L2 of a single HPV type, or as a mixture of several high-risk HPV types and administered utilizing electroporation, i.m. injection or gene gun. Serum was collected two weeks and 3 months after the last vaccination. Sera from immunized mice were tested for *in-vitro* neutralization titer, and protective efficacy upon passive transfer to naive mice and vaginal HPV challenge. Heterotypic interactions between L1 proteins of HPV6, HPV16 and HPV18 in 293TT cells were tested by co-precipitation using type-specific monoclonal antibodies.

**Results:**

Electroporation with L2 multimer DNA did not elicit detectable antibody titer, whereas DNA expressing L1 or L1+L2 induced L1-specific, type-restricted neutralizing antibodies, with titers approaching those induced by Gardasil. Co-expression of L2 neither augmented L1-specific responses nor induced L2-specific antibodies. Delivery of HPV L1 DNA via in vivo electroporation produces a stronger antibody response compared to i.m. injection or i.d. ballistic delivery via gene gun. Reduced neutralizing antibody titers were observed for certain types when vaccinating with a mixture of L1 (or L1+L2) vectors of multiple HPV types, likely resulting from heterotypic L1 interactions observed in co-immunoprecipitation studies. High titers were restored by vaccinating with individual constructs at different sites, or partially recovered by co-expression of L2, such that durable protective antibody titers were achieved for each type.

**Discussion:**

Multivalent vaccination via in vivo electroporation requires spatial separation of individual type L1 DNA vaccines.

## Introduction

Persistent infection by oncogenic human papillomavirus (HPV) drives the development of cervical cancer [1]. HPV infection also causes subsets of other cancers such as vulvar, vaginal, penile, anal, and oropharyngeal cancers [2], [3], [4]. The importance of preventing HPV infection drove the development of two commercial virus-like particle-based (VLP) vaccines, Gardasil® by MSD and Cervarix® by GSK, respectively. These two L1 VLP-based vaccines elicit robust type-restricted neutralizing antibodies that effectively inhibit HPV infection [5], [6], [7], [8], [9], [10], [11]. However, Gardasil® and Cervarix® each contain L1 VLP derived from only two high risk genotypes, HPV16 and HPV18, although Gardasil also contains L1 VLP derived from the two most common genotypes causing benign genital warts, HPV6 and HPV11. Since HPV16 and HPV18 cause 50% and 20% of all cervical cancers [12], [13], the two licensed vaccines are potentially able to prevent most but not all cases of cervical cancer because of the type-restricted immunity [14], [15]. However, HPV16 causes ∼90% of cases of HPV-associated vaginal, vulval, anal and oropharyngeal cancers, suggesting a distinct type distribution at these anatomic sites [2], [3], [4]. Passive transfer studies in animal models of HPV infection suggest that the type-restricted neutralizing antibodies induced by L1 VLP vaccination effect protection, although a role for cellular immunity has not been excluded [16]. The breadth of protection may be expanded by simply increasing the number of L1 VLP of different HPV genotypes, although this increases the cost and complexity of production. Merck is currently testing a nonavalent L1 VLP vaccine that targets the seven most common HPV genotypes found in cervical cancer and two types that cause most cases of genital warts [17].

The minor capsid protein, L2, harbors several conserved neutralizing epitopes at its amino terminus that elicits cross-protection among diverse HPV types [18], [19], [20], [21]. However, by comparison to L1 VLP, weaker immunogenicity is an obstacle L2 vaccine development [20], [22]. Several attempts have been made to enhance immunogenicity of L2 conserved epitopes and create a single vaccine protective against most high-risk HPV types. For example, L2 epitopes have been displayed repetitively by generating L2 multimer fusion proteins, or insertion into the immunodominant neutralizing epitope of VLPs of HPV and other viruses [23], [24], [25], [26].

Cost and the need for a cold chain are barriers to global implementation of HPV immunization. Unfortunately, 85% of cervical cancer cases occur in women in developing countries and even the tiered pricing for the two licensed vaccines is beyond the reach of many lower income countries [27]. The L2 multimer vaccine can be manufactured as a single protein in the E. coli system lowering its cost compared to multivalent L1 based vaccines produced in yeast or insect cells [28], [29], [30]. However, protein-based vaccines are prone to degradation at ambient temperature and typically require refrigeration such that development of heat-stable formulations is needed to facilitate implementation in low income and remote populations [30].

Naked DNA vaccines encoding vaccine antigens have several potential advantages. Production of DNA vaccines does not require culture, inactivation of infectious pathogens, and their purification from bacteria is well standardized and comparatively inexpensive [31]. Importantly, naked DNA can be readily stored at ambient temperature. Moreover, the antigenic structure of the vaccine antigen produced by DNA vaccination likely closely resembles the appropriate native structure with the correct post-translational modifications. Indeed, L1 expressed in E. coli does not form regular VLPs and requires in vitro disassembly and re-assembly [29], [32]. Furthermore, the DNA vector itself can have an adjuvant effect via its inherent immunostimulatory elements. Unmethylated CpG dinucleotide motifs can be sensed by Toll-like receptor (TLR)-9 [33], a microbial pattern recognition receptor (PRR), and trigger innate inflammatory responses [34], [35], [36]. DNA in the cytoplasm can be recognized and stimulate Absent In Melanoma 2 (AIM2) [37], and STimulator of IFN Genes (STING) pathways [38]. DNA vaccines also provide sustained antigen expression for a prolonged immune stimulation compared to the short half-life of protein antigens [39]. Despite many advantages over protein vaccines, low immunogenicity is a major shortcoming of DNA vaccines, and is believed to reflect inefficiency of delivery of the vaccine to the host nucleus.

There are several alternative modes of DNA administration that can overcome inefficient delivery. The gene gun provides ballistic delivery of gold particles coated with DNA to cells in the skin including professional antigen presenting cells, termed Langerhans cells [40], [41]. While the method is more efficient than i.m. injection, only a limited amount of DNA can be used due to technical issues. A second improved method of DNA delivery via in vivo electroporation elicits robust immune responses as a consequence of increased transfection of somatic cells and inflammation caused by localized cell death [42], [43], [44]. The potential of electroporation in clinical trials has recently been demonstrated with DNA vaccines targeting hepatitis B virus [45], HIV [46], [47] and HPV oncoproteins, E6 and E7 [48].

Here we show the potential of L1-expressing DNA vaccines administered with electroporation as a prophylactic vaccine. In addition, our results also demonstrate interference between L1 DNA vaccines administered at the same site with electroporation, likely reflecting co-assembly of different L1 into chimeric particles rather than immunologic competition. Finally, we find that this interference can be eliminated if L1 DNA vaccines of different HPV genotypes are spatially separated upon administration, or ameliorated if the cognate L2 proteins are co-expressed.

## Materials and Methods

### Ethics Statement

This study was carried out in strict accordance with the recommendations in the Guide for the Care and Use of Laboratory Animals of the National Institutes of Health. All animal studies were performed with the prior approval of the Animal Care and Use Committee of Johns Hopkins University (protocol MO08M19).

### Vaccine Preparation

Codon optimized capsid genes, L1 and L2, of HPV6, HPV16, HPV18, HPV26, and HPV51 were sub-cloned into double expression vector, pVITRO1-neo-mcs (Invivogen, San Diego CA) or pcDNA (Invitrogen, Carlsbad CA), for DNA vaccination. HPV16 L2 multimer expression construct encompassing residues 11−88 of 8 HPV types (L2×8) was sub-cloned into pcDNA 3.1 for mammalian expression, and L2 α11−88×8 polypeptide was produced, purified, and dialyzed as previously described [49]. All plasmids employed for the immunization were purified and endotoxins were removed with UltraClean® endotoxin free kit (Mo Bio, Carlsbad CA).

### Pseudovirus (PsV) Production

Codon optimized L1 and L2 capsid genes of HPV6, 11, 16, 18, 26, 31, 45, 51, were sub-cloned into double expression vector, pVITRO1-neo-mcs (Invivogen, San Diego CA). Pseudovirions (PsVs) were generated in 293TT cells following the standard PsV production protocol (http://home.ccr.cancer.gov/Lco/pseudovirusproduction.htm). Firefly luciferase expression plasmid was employed as a reporter for PsV infection in neutralization assays and for vaginal challenge studies.

### Immunoprecipitation

293TT cells grown in DMEM-10 medium were transfected with empty vector, a single expression vector for only HPV6, 16, or 18 L1, or mixture of L1 DNA of HPV6, 16, 18 in equal parts using transit 2020 (Mirus Bio LLC, Madison WI), and harvested in 48 hours. Cells were lysed with non-denaturing lysis buffer containing 20 mM Tris HCl pH 8, 137 mM NaCl, 10% (*v*/*v*) glycerol, 1% (*v*/*v*) Nonidet P-40, 2 mM EDTA, and a protease inhibitor cocktail (Roche). mAb H18.F8 [9] was added to whole cell lysate (0.5 mg) and tumbled end-over-end overnight at 4°C. Protein G Sepharose (GE healthcare, Waukesha WI) was added and mixed for an additional 4 hours at 4°C. Resins were harvested by centrifugation at 14,000×g at 4°C for 1 min and supernatants were discarded. Resins were washed three times with 1 ml lysis buffer, resuspended in 2× sample loading buffer, and boiled for 5 min.

### Western blot analysis

Western blotting was performed with standard protocols (http://www.abcam.com/ps/pdf/protocols/WB-beginner.pdf). Primary antibodies used were H6.C6, H16.O7, and H18.E20 [9], and secondary antibody used was HRP-goat anti-mouse IgG light chain (Jackson ImmunoResearch, West Grove PA).

### Vaccination

Groups (n = 5) of 3–4 week old female Balb/c mice were vaccinated s.c. three times at two week intervals with 25 µg of L2×8 multimer polypeptide formulated with alum (50 µg), or 50 µl of Gardasil, or i.m. with 10 µg of L2 11−88×8 multimer expression plasmid, or i.m. utilizing *in vivo* electroporation with PBS, 10 µg of L2×8 multimer expression plasmid in pcDNA 3.1, 2 µg or 10 µg of HPV6, 16, 18, 26, 51 L1 expression plasmid in pVITRO1-neo-mcs, 20 µg of HPV6, 16, 18 L1 expression plasmid in pVITRO1-neo-mcs, 10 µg of HPV11, 16, 18, 26, 31, 45, 51 L1+L2 expression plasmid in pVITRO1-neo-mcs, 20 µg of HPV6, 16, 18 L1+L2 expression plasmid in pVITRO1-neo-mcs, 10 µg of mixed (HPV 6, 16, 18, 26, 51 L1, 2 µg each) plasmids in pVITRO1-neo-mcs, 60 µg of mixed (HPV6, 16, 18 L1, 20 µg each) plasmids in pVITRO1-neo-mcs, 60 µg of mixed (HPV6, 16, 18 L1+L2, 20 µg each) plasmids in pVITRO1-neo-mcs, or 20 µg of HPV6, 16, 18 L1 expression plasmids in pVITRO1-neo-mcs at different sites. Blood samples were collected two weeks and three months after the last vaccination. Serum was separated by centrifugation at 2,000 *g* for 10 min at 4°C after the blood had congealed overnight at room temperature.

### Electroporation

Mice were injected with DNA in 30 µl water i.m. into the gastrocnemius muscle, or biceps femoris muscle of hind leg. A pair of electrode needles was inserted into the muscle flanking injection site and electrical pulses were delivered utilizing an ECM830 electroporation generator (BTX Harvard Apparatus company, Holliston MA). Eight pulses of 106 V each were delivered for 20 ms pulse duration at 200 ms intervals.

### ELISA

For analysis of antibody response against HPV16 L2 protein, microtiter plates were coated with L2 protein at 500 ng in 100 ul PBS/well overnight at 4°C, and blocked with PBS/1% BSA for 1 hour at 37°C. Plates were incubated with serum samples diluted 1 50 in PBS/1% BSA for 1 hour at 37°C. After 3 washes with washing buffer (0.01% *v/v* Tween 20 in PBS), HRP-sheep anti-mouse IgG diluted in 1% *w/v* BSA at 5000-fold was added to each well as a secondary antibody, and incubated for 1 hour at 37°C. After 3 further washes, 100 ul of ABTS solution, 2,2′ Azinobis [3-ethylbenzothiazoline-6-sulfonic acid], Roche, (Basel Switzerland) was added to each well for developing color and read by an ELISA reader, Benchmark Plus (Bio Rad, Hercules CA) at 405 nm.

### *In vitro* Neutralization Assays

Serum samples (4 µl) were serially diluted two-fold in culture media, and mixed with 0.03 µl of HPV PsV carrying luciferase reporter plasmid. Mixtures were incubated at 37°C for two hours, added to 3×10^4^ of 293TT cells, and incubated at 37°C for 72 hours. Cells were washed with 1× PBS, lysed with 30 µl of Cell Culture Lysis Reagent (Promega, Madison WI) for 15 min at room temperature on a rocking platform. Lysates were transferred to 96-Well black plate, and luciferase activity was measured by GloMax®-Multi Detection System (Promega, Madison WI) after adding 50 ul of luciferin substrate (Promega, Madison WI) to each well.

### Vaginal Challenge Studies

Mice were subcutaneously injected with 3 mg of medroxyprogesterone (Depo-Provera, Pfizer, New York NY) four days before vaginal challenge to synchronize their estrus cycles. Viral challenge was performed by delivery of 10 µl of HPV PsV (HPV6: 135 billion, HPV16: 189 billion particles in total) mixed with 10 µl of 3% carboxymethyl cellulose (CMC) into the vagina before and after cytobrush treatment (15 rotations, alternating directions) while the mice were under isoflurane anesthesia. Three day after challenge, mice were anesthetized by isoflurane, and 20 µl of luciferin substrate (7.8 mg/ml, Promega, Madison WI) was delivered into the vaginal vault before imaging. Bioluminescence was acquired for 10 min with a Xenogen IVIS 100 (Caliper Life Sciences, Hopkinton MA) imager, and analysis was accomplished with Living Image 2.0 software. For imaging of passively immunized mouse groups, mice were injected i.v. with 20 µl of serum one day before vaginal challenge.

### Statistical analysis

Exploratory statistical analyses are performed to analyze the observed titer data. Square-root data transformations were used to achieve normality in residuals for titer data. One-way ANOVA and pair wise multiple comparisons with Bonferroni adjustment were performed using SAS 9.3. When the overall significance test is not significant, the pair wise comparisons are not conducted. To claim significance, we use alpha level 0.1 for pair wise comparisons. The homogeneity of variances is examined with Levene's test.

## Results

### *In vivo* electroporation with HPV L1 DNA vaccines elicits robust type-restricted neutralizing antibody titers in mice

**Figure 1:**
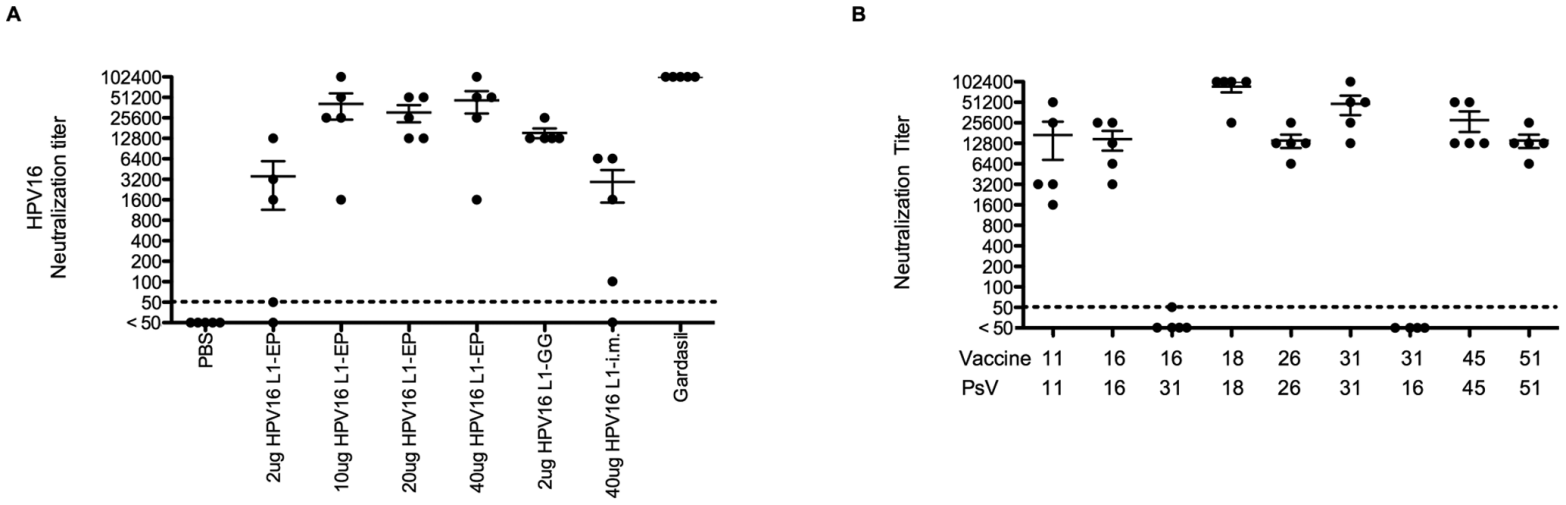
Impact of DNA vaccine dose and delivery method on the induction of type-restricted neutralizing antibodies. (A) Balb/c mice were vaccinated three times at two week intervals with HPV16 L1 DNA expression vector via i.m. injection alone (40 µg)(i.m.), i.m. injection and *in vivo* electroporation (0, 2, 10, 20 or 40 µg)(EP) or i.d. ballistic delivery on gold particle via gene gun (2 µg)(GG). Vaccination with Gardasil s.c. was included as a positive control. Serum samples were collected two weeks after the third vaccination, and tested for in vitro neutralization assay against HPV16. (B) Mice were vaccinated with DNA vector expressing L1+L2 of the genotypes indicated (see line labeled ‘Vaccine’) i.m. utilizing electroporation. Neutralizing antibody titer against PsV of the indicated genotypes (see line labeled ‘PsV’) was measured for sera harvested two weeks after the last vaccination.

Vaccination of rabbits with naked DNA expressing CRPV L1 either via i.m. injection or ballistic delivery on gold beads (gene gun) protects rabbits from experimental viral challenge [50], [51]. The initial enthusiasm for naked DNA vaccination based on animal data has been tempered by a low efficiency of delivery by *i.m.* injection of patients. However, recent clinical studies suggest that *in vivo* electroporation of naked DNA vaccines can induce robust humoral responses in patients [47], [48], [52], [53], [54]. Furthermore, codon optimization appears to be important for robust expression of the HPV capsid proteins [55], [56], [57]. Therefore we sought to determine whether a strong neutralizing antibody response could be elicited in mice with codon optimized HPV16 L1 capsid gene-expressing naked DNA constructs utilizing i.m. injection as compared to i.m. injection with *in vivo* electroporation, or i.d. delivery via gene gun. We utilized HPV pseudovirion infection of 293TT cells to examine neutralization by serum antibody as a surrogate of wart-derived virions infecting primary human keratinocytes [58]. Vaccination of mice three times at 2 week intervals with 40 µg of HPV16 L1 DNA by i.m. injection resulted in a weak HPV16 neutralizing antibody response as compared to s.c. administration of 1/10^th^ of a human dose of Gardasil for which the serum titer was 2 log_10_ greater (Figure 1A). The response was greater when this HPV16 L1 DNA vaccine was administered i.d. via gene gun at a dose of 2 µg (the maximum that can be applied by this method), but the titer was still a log_10_ below that obtained with Gardasil. When the HPV16 L1 DNA vaccine was injected i.m. followed by in vivo electroporation, doses of 10 µg or greater elicited an HPV16 neutralizing antibody titer approaching that of Gardasil, whereas the response to the 2 µg dose was weak. A one-way analysis of variance (ANOVA) shows that there is significant difference among 2 µg, 10 µg, 20 µg and 40 µg HPV16 L1 electroporation (EP) groups (p-value = 0.0347). With Bonferroni multiple comparison adjustment, at α level 0.1, we find a significant difference between 2 µg and 10 µg HPV 16 L1-EP groups (adjusted p-value 0.0991) and between 2 µg and 40 µg HPV 16 L1-EP groups (adjusted p-value = 0.0582). A significant difference is also detected between 2 µg HPV16 L1-GG and 40 µg HPV 16 L1-EP groups (p-value = 0.0031).

### *In vivo* electroporation with L1 only or L1+L2 DNA vaccines elicits a similar antibody response

The co-expression of the minor capsid protein L2 with L1 can enhance the efficiency of VLP assembly [59]. Therefore, we explored whether a higher titer antibody response could be achieved upon vaccination with a DNA vaccine expressing both L1 and L2 versus L1 alone. However, vaccination three times i.m. using in vivo electroporation with 10 µg of a DNA vaccine expressing HPV16 L1+L2 elicited a similar neutralizing antibody titer to HPV16 L1 alone. Further, vaccination three times i.m. using in vivo electroporation to deliver 10 µg of a DNA vaccine expressing L1+L2 derived from HPV11, HPV18, HPV26, HPV31, HPV45 or HPV51 each elicited a similar neutralizing antibody titer against PsV of the same type utilized for vaccination (Figure 1B). The titers of neutralizing antibodies induced upon vaccination three times i.m. using in vivo electroporation to deliver 10 µg of a DNA vaccine expressing L1+L2 were similar to, but still lower than those elicited by Gardasil. Since the responses to electroporation of 10 µg of a DNA vaccine expressing L1+L2 appeared more variable than for Gardasil,the homogeneity of variances among L1 DNA EP, L2×8 Protein and Gardasil groups was examined with Levene's test. While the difference is not significant (p-value = 0.0556), this may reflect the rather limited sample size (5 mice in each group).

**Figure 2:**
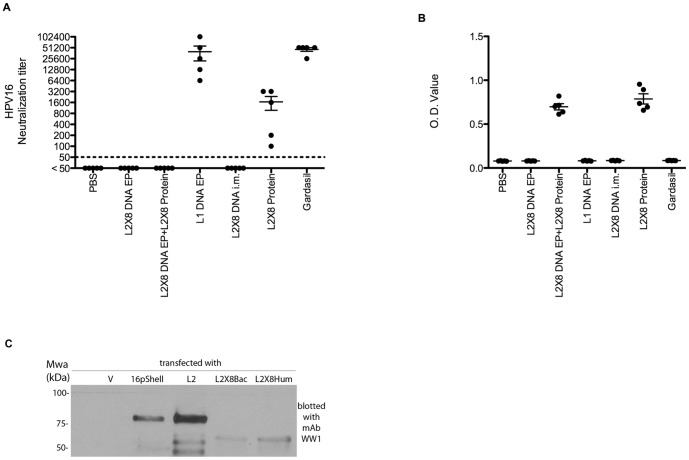
Neutralizing antibody titer and antibody response of sera from mice vaccinated with L1 or L2×8 delivered as protein or a DNA vaccine via electroporation. Balb/c mice were vaccinated three times at two week intervals with PBS, 10 µg L2×8 DNA vaccine i.m. with electroporation three times, or twice utilizing 10 µg HPV16 L1 DNA vaccine i.m. with electroporation followed by a single boost with 25 ug L2×8 protein in alum s.c., 10 µg HPV16 L1 DNA vaccine i.m. with electroporation three times, three times with 10 µg L2×8 DNA vaccine i.m., or 25 ug L2×8 protein in alum s.c., or Gardasil s.c.,,. Serum samples were collected two weeks after the third vaccination, and were tested for in vitro HPV16 neutralization titer (A) and antibody response to HPV16 L2 (B) as measured by ELISA. (C) To assess relative levels of expression, 293TT cells were transfected with no plasmid, HPV16 L1+L2 DNA in pShell, full length HPV16 L2 DNA in p16L2h, bacterial codon optimized L2×8 DNA in pcDNA, or human codon optimized L2×8 in pcDNA. 293TT cells were lysed two days after transfection. Western blotting was performed with lysate samples using a monoclonal antibody to HPV16 L2 17–36.

L2 contains cross-neutralizing epitopes, and therefore we tested whether in vivo electroporation of mice with 10 µg of the DNA vaccine expressing HPV16 L1+L2 induced antibodies that cross-neutralized PsV of HPV31, the genotype most closely related to HPV16, or *vice versa*. However, no significant cross-neutralization was observed between these two types (Figure 1B), and no L2-specific antibody response was observed (despite robust L2 expression, Figure 2C) indicating that L2 is not immunogenic in the context of the capsid. These findings suggest that a multivalent L1-based DNA vaccine approach or vaccination with L2 (in the absence of L1) would be necessary to generate a broadly protective response.

### *In vivo* electroporation with an L2 multimer DNA vaccine failed to elicit neutralizing antibodies

Prior studies by Hitzeroth *et al* suggest that i.m. vaccination of mice with 100 µg DNA expressing codon-optimized full length HPV16 L2 induces a T cell response but only a very weak antibody titer, whereas vaccination with the amino terminus of L2 may be more immunogenic [60], [61]. Vaccination of mice with a multimer peptide comprising L2 residues 11−88 amino acids derived from 8 HPV types (L2×8) induced broadly neutralizing antibodies, although at a titer ∼30-fold lower than for L1 VLP [49]. Therefore we compared the HPV16 neutralizing antibody titers induced upon vaccination via *in vivo* electroporation with codon-optimized HPV16 L1 DNA versus L2×8 multimer DNA. While the L1 DNA construct elicited very high neutralization titers approaching those induced by Gardasil vaccination (Figure 2A), L2×8 multimer DNA vaccination failed to induce neutralizing antibody against HPV16 pseudovirions, even with a single L2×8 protein in alum boost (although the latter did induce an L2-specific response detectable by ELISA with a titer of 400). Vaccination with L2×8 multimer DNA failed to induce a neutralizing antibody response either with or without electroporation, and whether the codon optimization of the L2×8 construct was biased for bacterial expression or mammalian expression (not shown). Similar levels of expression of L2×8 protein were detected in 293TT cells 48 h post transfection with the L2×8 constructs codon optimized for bacterial or mammalian cell expression (Figure 2C). The levels of L2×8 were substantially lower than for full length codon-optimized HPV16 L2 and there was evidence of a greater extent of degradation, possibly contributing to the poor immune response (Figure 2C).

Vaccination with antigen delivered first via DNA vaccine and then boosting with protein has been suggested to enhance humoral immunity as compared to DNA vaccination or protein alone [62], [63]. We tested the sera of mice vaccinated twice with L2×8 multimer DNA followed by a third immunization of L2×8 multimer peptide mixed with alum adjuvant. Surprisingly, we were not able to detect an HPV16 neutralizing antibody titer with the L2×8 multimer DNA vaccine prime and protein boost combination regimen (Figure 2A), although antibody response to full length HPV16 L2 was detected (Figure 2B) with an ELISA titer of 400. In contrast, vaccination three times with L2×8 protein in alum elicited L2-specific antibody (ELISA titer of 12,800) and significant titers of HPV16 neutralizing antibody (a mean titer of 1600, Figure 2A), although the latter is ∼30-fold lower than for L1 DNA vaccination or Gardasil. This suggests that even vaccination twice with L2×8 multimer DNA was inadequate for priming a neutralizing antibody response prior to a single L2×8 protein boost, and this may reflect poor expression, protein instability, insensitivity of the neutralization assay, weak immunogenicity for L2×8 vaccination via DNA vector, and/or the preferential induction of antibody to non-neutralizing L2 epitopes when using this immunization regimen.

### Vaccination with a mixture of L1 DNAs of multiple HPV types exhibits interference

Since L1-based DNA vaccine electroporation successfully induced a robust but type-restricted neutralizing antibody response, we evaluated the potential for a multivalent vaccine in which L1 DNAs of several HPV types are mixed prior to i.m. injection and *in vivo* electroporation. Our initial study suggested that a 2 µg dose of HPV16 L1 DNA vaccine induces a suboptimal neutralizing antibody response, but 10 µg produces the maximal response. To examine if the 2 µg L1 DNA vaccine dose provided a consistent response regardless of HPV type, and whether the response to a pentavalent vaccine would be additive or synergistic, we performed a pilot experiment in which five different DNA vaccines expressing L1 of HPV6, HPV16, HPV18, HPV26, and HPV51 respectively were mixed (2 µg each) and delivered i.m. by electroporation for comparison to vaccination with each type singly at doses of 2 µg or 10 µg. HPV6, HPV16 and HPV18 are each members of different papillomavirus species (α10, α9, and α7 respectively), whereas HPV26 and HPV51 are both members of the α5 species. The latter two types were included to determine if interference in the response to mixtures of L1 expression constructs occurred intra-species or inter-species or both [64]. After three immunizations, mice vaccinated with the pentavalent L1 DNA mixture exhibited distinct neutralizing antibody responses compared to mice vaccinated with L1 DNA of single HPV type (Table 1). The 2 µg dose of a single L1 DNA vaccine gave an inconsistent response for HPV16 and between different HPV types, whereas the response to 10 µg was consistent across all 5 types tested. The variable response at the 2 µg dose of a single L1 DNA vaccine did not reflect L1 expression level alone. Indeed, the relative number of particles produced using these codon-optimized L1 genes to produce pseudovirions in 293TT cells, as estimated by Coomassie-stained SDS-PAGE gel studies of purified pseudovirions and normalized to HPV16 are: HPV6 L1: 15.5%, HPV16 L1: 100%, HPV18 L1: 1%, HPV26 L1: 119%, HPV51 L1: 147%. Thus despite significantly lower production of particles by HPV6 and HPV18, both of these constructs elicit a robust neutralizing antibody response. Serum from the L1 DNA mixture group showed no detectable neutralizing titer against HPV6 and HPV16 PsVs, the expected titer for HPV51 but an increased titer against HPV18 and HPV26 PsVs (Table 1). This finding suggested that the DNA constructs may not act independently when mixed together and that increasing the dose of L1 DNA vaccine from 2 µg to 10 µg improves the level and consistency of the neutralizing antibody response.

**Table 1:** Neutralizing antibody titers induced upon vaccination of mice with a pentavalent HPV L1 DNA vaccine.

Vaccine	Dose ( µg)	HPV6 IVNT	HPV16 IVNT	HPV18 IVNT	HPV26 IVNT	HPV51 IVNT
vector	10	ND	ND	ND	ND	ND
HPV6 L1	2	ND				
	10	6,400				
HPV16 L1	2		ND			
	10		6,400			
HPV18 L1	2			50		
	10			51,200		
HPV26 L1	2				1,600	
	10				6,400	
HPV51 L1	2					50
	10					12,800
HPV6,16,18,26, and 51 L1	2,2,2,2,2	ND	ND	6,400	6,400	50
Gardasil	0.1 mL	102,400	102,400	102,400	ND	ND

Balb/c mice were vaccinated i.m. three times at two week intervals with a mixture of L1 vectors of HPV6, HPV16, HPV18, HPV26, and HPV51 (2 µg each), L1 vector of single HPV type (2 µg or 10 µg) utilizing electroporation. Gardasil vaccination was included as a positive control. Sera from 5 mice were pooled together, and *in vitro* neutralizing antibody titer (IVNT) was measured with HPV6, HPV16, HPV18, HPV26, and HPV51 PsVs. ND: Not detected at 1:50.

### Spatial separation of L1 DNA vaccines of different types, but not L2 co-expression, fully restores independent neutralizing antibody responses upon multi-type vaccination

**Figure 3:**
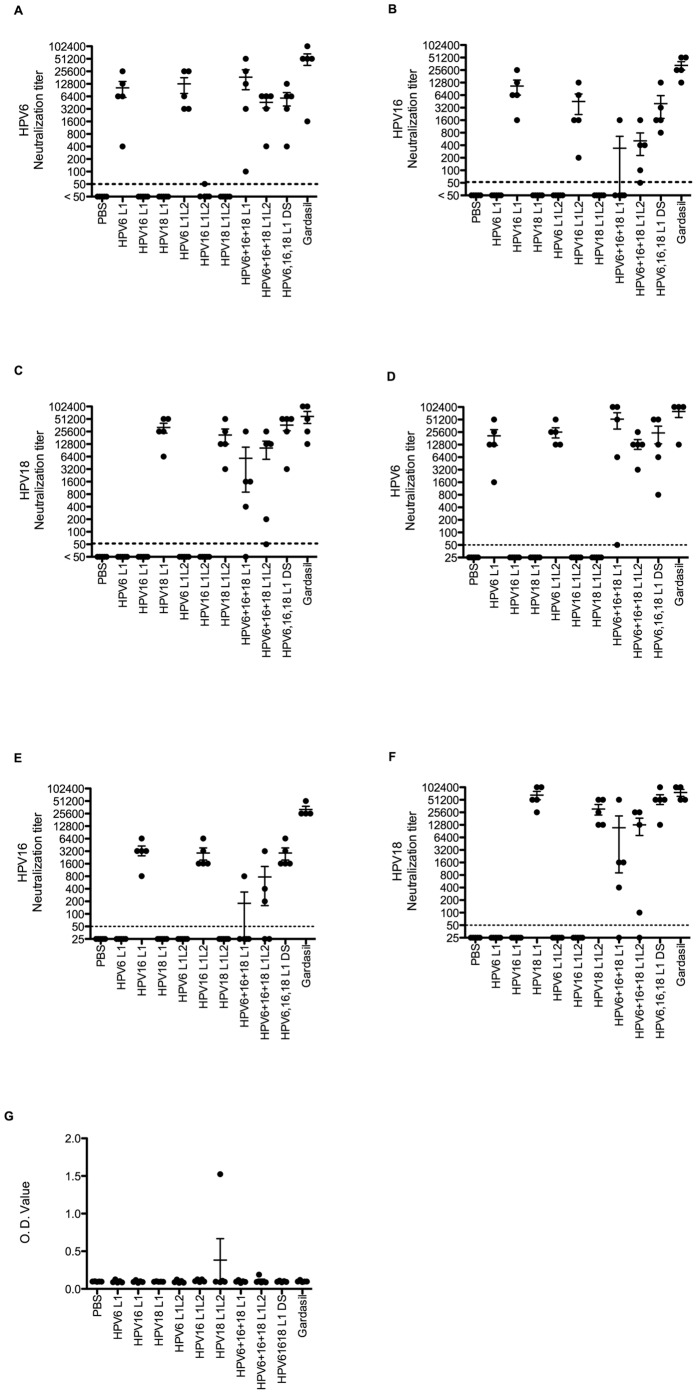
A comparison of antibody responses of mice vaccinated with DNA expressing L1 or L1+L2 of HPV6, 16, or 18, either singly or together at the same or different sites. Balb/c mice were vaccinated i.m. with electroporation three times at two week intervals with 20 µg each of DNA expressing L1 or L1+L2 of HPV6, HPV16, and HPV18, either individually, or together at the same site or each at a different site (DS), or s.c. with Gardasil. Serum samples were harvested at two weeks after the third vaccination (A–C) or 3 months after the third vaccination (D–F), and neutralizing antibody titers were measured with HPV6 (A,D), HPV16 (B,E), or HPV18 PsV (C,F). Antibody response to L2 was measured by ELISA against full length HPV16 L2 with serum samples collected two weeks after the third vaccination (G).

In a follow-up study (Figure 3), several changes were made in order to address interference when L1 DNA vaccines are mixed. First, the dose of each L1 DNA was increased from 2 µg to 20 µg to enhance the level and consistency of the neutralizing antibody response, and the number of HPV types decreased from five to three (HPV6, HPV16 and HPV18) to minimize the potential for interference and because vaccination with Gardasil suggests limited or no immunologic intereference in the antibody responses to L1 VLPs these three types (Table 1). Furthermore, these types are the most common causing genital warts (HPV6) and squamous cell (HPV16) and adenocarcinoma (HPV18) of the uterine cervix, suggesting their likely inclusion in future vaccines as compared with HPV26 and HPV51 that are infrequently found in cervical cancer [65]. It is possible that the immunologic interactions between the L1 vaccines when administered as a mixture reflect heterotypic binding and inappropriate co-assembly and/or immunologic dominance of particular construct(s). Therefore, to prevent co-assembly of L1 of different types into a chimeric VLP, we tested the impact on the humoral response of administering the three L1 DNA constructs each at a different site (HPV6 L1 DNA was injected into the left biceps femoris muscle, HPV16 L1 DNA was injected into the right gastrocnemius muscle, and HPV18 L1 DNA was injected into the left gastrocnemius muscle) versus mixed together and delivered at the same site. In addition, L2 exhibits some type-restriction for interaction with L1 [66], [67], [68], and facilitates VLP assembly by ∼4-fold [69]. Therefore, mice were vaccinated with a mixture of three DNA vaccines expressing L1+L2 of HPV6, HPV16 and HPV18 based upon the hypothesis that the presence of the cognate L2 would both increase the assembly of VLPs four-fold and the specificity of co-assembly with the homotypic L1, thus limiting heterotypic VLP production. Two weeks after three vaccinations i.m. with the DNA constructs indicated, the *in vitro* neutralization titer of the serum of each mouse was tested against HPV6, HPV16 and HPV18 PsV. Vaccination with 20 µg of each L1 DNA vaccine individually induced a robust homotypic neutralizing antibody response and an equivalent titer was observed when utilizing DNA vaccines expressing L1+L2 (Figure 3A–C) as the Bonferroni adjusted pair wise comparisons are not significant in all cases at alpha level 0.1. Despite reducing the number of HPV L1 DNAs to three types, (HPV6, HPV16, and HPV18), there was still some interference in production of neutralizing antibody, most noticeably for HPV16 (Figure 3B) for which ANOVA analysis results show that there is significant difference among groups (p-value = 0.0030), to a lesser extent for HPV18 (Figure 3C) (p-value = 0.0243), but not significantly for HPV6 (Figure 3A) (p-value = 0.5577). As seen in figure 3A, the HPV16 neutralizing serum antibody titer induced by vaccination with HPV16 L1 alone was significantly higher than when the HPV6, HPV16 and HPV18 L1 constructs were mixed together and injected at the same site (the Bonferroni adjusted t-test p = 0.0038), but not when given at different sites (p = 0.6893) or when L2 was co-expressed in the constructs (p = 0.7567). This pattern was also observed for sera harvested 3 months post vaccination (Figures 3D-F) as ANOVA analysis results show that there is again significant difference among groups for HPV16 (Figure 3E, p-value = 0.0005), and HPV18 (Figure 3F, p-value = 0.0027), but not for HPV6 (Figure 3D, p-value = 0.6497). Specifically, in figure 3E, the HPV16 neutralizing serum antibody titer induced 3 months after vaccination with HPV16 L1 alone was significantly higher than when the HPV6, HPV16 and HPV18 L1 constructs were mixed together and injected at the same site (the Bonferroni adjusted t-test p = 0.0394), but not when given at different sites or when L2 was co-expressed in the constructs. Importantly, when the three L1 DNA vaccines were each administered at a physically separate site, robust homotypic neutralizing antibody titers were observed, consistent with those obtained when administering each construct alone for all three virus types. This observation suggests that the interference does not reflect immunologic competition, but rather suggests that co-expression of L1 of HPV6, HPV16 and HPV18 in the same cells might result in aberrant VLP assembly as a consequence of heterotypic binding. While vaccination at different sites resolved interference completely, electroporation of mice with a mixture of three DNA vaccines co-expressing both L1 and L2 of HPV6, HPV16 and HPV18 only partially reduced interference in the responses to HPV16 and HPV18 (Figure 3B and C). This phenomenon was preserved when analyzing sera harvested at 3 months after the final vaccination (Figures 3D–F). We also tested whether vaccination with L1+L2 DNA of a single type or a mixture of three types can generate an L2-specific antibody response but no consistent antibody response against HPV16 L2 was observed (Figure 3G). These results imply that formation of chimeric VLP still occurs when the valency of the L1 multitype vaccination is reduced, and this problem can be ameliorated by co-expression of the cognate L2 proteins, but is eliminated by vaccinating at different sites.

### Electroporation of L1 DNA vaccines induces type-restricted protective antibody responses

**Figure 4:**
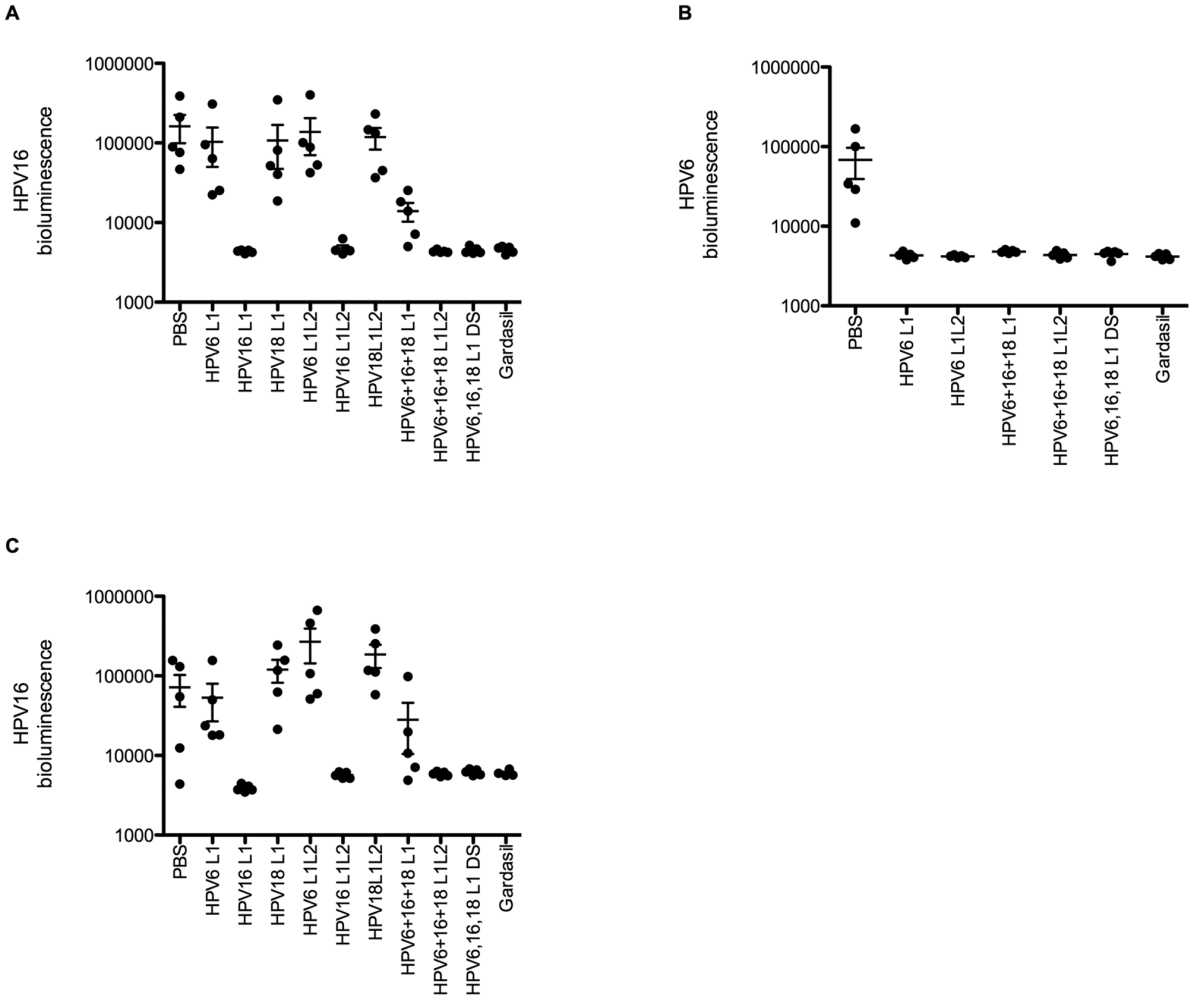
Comparison of protective antibody responses. Balb/c mice were vaccinated i.m. with electroporation three times at two week intervals with 20 µg each of DNA expressing L1 or L1+L2 of HPV6, HPV16, and HPV18, either individually, or together at the same site or each at a different site, or s.c. with Gardasil. Serum samples were collected two weeks (A,B) and three months (C) after the last vaccination to test their protective efficacy *in vivo* and *in vitro*. Naïve Balb/c mice (5 per group) were passively immunized i.v. with 20 µl of pooled serum. Mice were challenged intra-vaginally with HPV16 (A,C) or HPV6 (B) PsV carrying a luciferase reporter construct. Three days later, luciferin was administered intra vaginally, and bioluminescence imaging was performed.

As the titers of neutralizing antibodies induced upon vaccination three times i.m. using *in vivo* electroporation to deliver 20 µg of an L1 DNA vaccine were similar to, but still lower than those elicited by Gardasil, we therefore tested whether they are still sufficient to protect against vaginal challenge with the homologous type HPV. Although HPV does not produce disease in mice, the host restriction in HPV infection is determined after delivery of the viral genome to the nucleus, and thus infection of the vaginal tract of mice with HPV pseudovirions carrying a luciferase reporter can be detected by imaging bioluminescence [70]. Passive transfer of 20 µl of serum from mice vaccinated i.m. three times with *in vivo* electroporation to deliver 20 µg of DNA vaccine expressing either L1 only or L1+L2 completely protected naïve mice from experimental vaginal challenge with the homologous genotype (Figure 4A). This was also shown for HPV6 (Figure 4B), and the protection was maintained when using sera harvested at 3 months post vaccination for the passive transfer study (Figure 4C). Thus the data suggest that the neutralizing antibody titers induced by *in vivo* electroporation with an L1 DNA vaccine, although lower than for Gardasil, are sufficient for complete protection against vaginal challenge by the vaccine type.

### L2 co-expression is sufficient to restore protection using multivalent L1-based DNA vaccination

The host restriction in HPV infection is determined after delivery of the viral genome to the nucleus, and thus infection of the vaginal tract of mice with HPV pseudovirions carrying a reporter gene such as luciferase whose expression can be detected by imaging bioluminescence [70]. This provides a useful model to examine in vivo protection of naïve mice after passive transfer of neutralizing antibodies [71], [72]. Passive transfer of 20 µl of serum of mice electroporated i.m. with a mixture of three DNA vaccines expressing L1 of HPV6, HPV16, and HPV18 provided complete protection against intra-vaginal challenge with HPV6 PsV (Figure 4B). In contrast, when 20 µl of serum of mice electroporated i.m. with a mixture of three DNA vaccines expressing L1 of HPV6, HPV16, and HPV18 was injected into naive mice, significantly weaker protection against intra-vaginal challenge with HPV16 PsV was observed (Figure 4A). Interestingly, the sera from mice that were electroporated i.m. with a mixture of three DNA vaccines expressing both L1 and L2 of HPV6, HPV16, and HPV18, were completely protective by passive transfer. This finding suggests that although the co-expression of L2 does not completely recover the humoral response obtained by vaccination with a single L1 DNA vaccine, nevertheless the titers induced are sufficient for complete protection upon passive transfer of 20 µl of serum (which corresponds to ∼1:50 dilution in the mouse). As expected, passive transfer of sera from mice electroporated i.m. at different sites with the three DNA vaccines expressing L1 of HPV6, HPV16, and HPV18, were also completely protective against HPV16 challenge. Importantly, these phenomena were consistent when testing for protective capacity against vaginal HPV16 challenge of naïve mice after passive transfer of 20 µl of sera obtained three months after active vaccination (Figure 4C). These data suggest that the protective responses elicited by multivalent vaccination are durable if the L1 DNA vaccines expressing individual types are injected in different locations or L2 is co-expressed with each L1.

### Immunologic interference is associated with heterotypic interactions between L1 proteins

**Figure 5:**
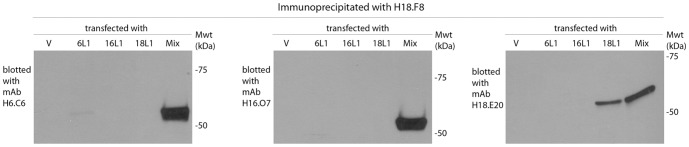
Interaction between HPV18 L1 with L1 of both HPV6 and HPV16. To test for interaction between L1 proteins of different HPV genotypes, 293TT cells were transfected with empty DNA vector (V), or vector expressing HPV6 L1, HPV16 L1, or HPV18 L1 individually (6L1, 16L1, 18L1, respectively), or all three together (Mix). Two days later, the cells were harvested and lysed. HPV18 L1 was immune-precipitated from lysates using the conformationally-dependent and neutralizing monoclonal antibody H18.F8. The presence of HPV6 L1, HPV16 L1 and HPV18 L1 in the immune-precipitates was detected by Western blotting with H6.C6 (left panel), H16.O7 (middle panel), and H18.E20 (right panel) respectively.

The reduction in neutralizing responses observed when the DNA vaccines expressing L1 of different types are injected into the same site but not different sites suggests that it is direct interaction between L1 of different types rather than immunologic competition that this responsible for this interference. It has been previously described that HPV6 and HPV16 L1 subunits co-assemble together generating hybrid VLPs in yeast [73] and co-expression of either HPV11 or BPV1 L1 with HPV16 L1 reduces the assembly of HPV16 L1 VLPs [74]. In addition, it is known that neutralizing epitopes on VLPs are conformationally-dependent and type-restricted [75], [76], [77]. Therefore, to test for direct interaction between L1 of different HPV types, 293TT cells were transfected with the three DNA vaccines expressing L1 of HPV6, HPV16, and HPV18 either individually or simultaneously, and immunoprecipitation experiments were performed on cell lysates using the H18.F8 monoclonal antibody that recognizes a conformational and type-specific HPV18 L1 epitope [78]. The presence of HPV6 L1, HPV16 L1 and HPV18 L1 in the immunoprecipitates was detected by Western blot analysis with type-restricted monoclonal antibodies H6.C6, H16.O7 and H18.E20 [78], respectively (Figure 5). H18.F8 immunoprecipitated HPV18 L1, but not HPV6 L1 or HPV16 L1 from lysates of cells transfected with only a single L1 DNA vaccine (Figure 5). By contrast, H18.F8 immunoprecipitated HPV6 L1, HPV16 L1 and HPV18 L1 from the lysates of cells co-transfected with the three DNA vaccines expressing L1 of HPV6, HPV16, and HPV18 L1. These results demonstrate that HPV18 L1 can bind to both HPV6 L1 and HPV16 L1.

## Discussion

L1 DNA vaccines have potential as an alternative prophylactic HPV vaccine that is simple and inexpensive to produce and heat stable, properties that would facilitate widespread immunization in low resource and remote settings. However, delivery of DNA vaccines *in vivo* has been limited by the efficiency of transfection *in vivo*. Several approaches including ballistic delivery via gene gun, *in vivo* electroporation devices, and tattooing have been developed to improve *in vivo* delivery. Here, we have tested two of these approaches previously tested clinically for DNA vaccine delivery; gene gun and *in vivo* electroporation. While gene gun vaccination demonstrated strong neutralizing antibody responses with only 2 µg of HPV16 L1 DNA vaccine, this was the highest amount of DNA that could be loaded onto the gold particles. The *in vivo* electroporation approach required 10 µg of HPV16 L1 DNA vaccine to achieve a consistent and robust neutralizing antibody response (that was not significantly higher with 20 µg or 40 µg doses), and these titers were higher than for 2 µg maximal dose of HPV16 L1 DNA vaccine delivered via gene gun. Although electroporation clearly enhances the efficiency of *in vivo* transfection of cells with the DNA vaccine, it may also trigger local inflammation, cell death and recruitment of immune cells to enhance the immune response [42], [43], [44]. While L1 DNA vaccination clearly has potential as a heat stable and low cost vaccine, a requirement for *in vivo* electroporation would raise the cost and complexity of vaccination because of the need for an electroporation device and access to electricity. Delivery via a self-contained gene gun device like PD-10 is another option, but the DNA dose is much more limited than for *in vivo* electroporation.

Hitzeroth *et al* observed a very weak (titer of 1:50) and non-neutralizing antibody response to vaccination of mice i.m. twice with 100 µg of DNA expressing full length codon-optimized HPV16 L2 [79]. The L2×8 DNA vaccination failed to elicit a detectable neutralizing antibody titer (Figure 2). A possible reason for the lack of response is poor expression by the L2×8 construct as compared with full length HPV16 L2 is incorrect codon optimization resulting in poor expression. However, when we re-optimized codon usage in the L2×8 construct for mammalian expression and compared expression levels to the previous bacterial codon optimized construct there was no significant improvement in expression. There are negative transcription and translation regulating sequences in the L2 gene, and the L2×8 DNA constructs may therefore still contain 8-fold more negative regulatory sequences than HPV16 L2, even after the nucleotide sequence changes resulting from codon optimization. In addition, recent studies suggest the presence of a transmembrane-like domain within this region of L2 that may limit expression and/or antigen release [80]. The L2×8 protein exhibited extensive degradation when expressed in mammalian cells (Figure 2C), which may also contribute to its low level and immunogenicity. Finally, it is clear that L2×8 is substantially less immunogenic than L1 VLP as the L2×8 protein vaccine induces ∼30-fold lower neutralizing antibody titers than Gardasil [49].

Previously it was shown that HPV6 L1 and HPV16 L1 can co-assemble forming hybrid VLP in yeast [73] and that co-expression of either BPV1 or HPV11 L1 with HPV16 L1 reduces HPV16 L1 VLP assembly in 293T cells [74]. We also showed that HPV18 L1 binds to both HPV6 L1 and HPV16 L1 when co-expressed in 293TT cells (Figure 5). The interaction between L1 of different HPV types, i.e. heterotypic binding, likely reflects the high degree of sequence conservation of the internal L1 structure, and has a deleterious effect upon multivalent L1 DNA vaccination. Indeed, mice vaccinated with a mixture of three DNA vaccines expressing L1 of HPV6, HPV16 and HPV18 showed relatively reduced protection against HPV16 compared to the group vaccinated with single HPV16 L1 DNA. This was also the case for HPV18 L1, but not for HPV6 L1. Thus the interference was inconsistent, possibly reflecting different L1 expression levels and affinity for heterotypic L1 interaction. Vaccinating at different sites completely restored the induced neutralization titer to the levels obtained when vaccinating with a single construct (Figures 3 and Figure 4). This observation suggests that spatial separation of individual type L1 DNA vaccines during vaccination prevents the formation of heterotypic L1 interactions and aberrant assembly of chimeric VLP. Three HPV types were used in our experiments, but more HPV types covering most of oncogenic types could potentially be delivered by a multi-microneedle injector to spare the patient from receiving multiple injections at each visit.

The co-expression of the cognate L2 in each construct is a possible alternative to spatial separation of the L1 DNA vaccines of different genotypes during multivalent vaccination. While this approach enhanced the neutralizing antibody responses relative to a mixed multivalent L1 DNA vaccine, it did not achieve in all cases the titers obtained upon vaccinating with a single type or spatial separation with multivalent L1 DNA vaccination (Figure 3). Nevertheless, the titers achieved with a mixed multivalent L1+L2 vaccine were completely protective against vaginal HPV16 challenge upon passive transfer to naïve mice with 20 µl of serum whereas antiserum from mice administered with the mixed multivalent L1 vaccine was only partially protective (Figure 4). This protection data is consistent with the in vitro neutralization titers and suggests that only a low neutralizing antibody titer is required for complete protection. Further the protective response to the electroporation of the mixed multivalent L1+L2 vaccine was durable at 3 months after the final vaccination, suggesting that co-expression of L2 might be an alternative to administering each construct at a different site.

Both of the licensed HPV vaccines utilize an alum-based adjuvant and Cervarix also includes a TLR4 agonist, MPL. Naked DNA vaccines, delivered appropriately, can potentially activate TLR9 via CpG islands as well as cytoplasmic DNA sensors such as DAI and AIM2 to enhance the immune response. However, we have not examined L1 DNA vaccination with alum and utilizing electroporation. Previously, it was found that mixing a hepatitis B DNA vaccine with aluminum phosphate improved the antibody titers ∼10-fold relative to DNA alone injected i.m. without electroporation [81]. While the titers of neutralizing antibody induced by L1 DNA vaccines might be improved with adjuvant and utilizing electroporation to reach the levels produced by Gardasil, it is clear that the L1 DNA vaccines elicit completely protective responses. Passive transfer of 20 µl of serum from either L1 DNA or Gardasil vaccinated mice rendered naïve animals immune to vaginal challenge. This corresponds to a ∼1:50 dilution in the mouse, indicating a robust response. Furthermore, while the studies demonstrate that neutralizing antibody is sufficient to mediate protection, it does not rule out a contribution of L1-specific T cell immune responses to protection in actively vaccinated mice. Further study is warranted to determine the relevance of L1-specific T cell immune responses to preventing HPV infections and the relative levels induced by L1 VLP protein and L1 DNA vaccination.

Safety issues and practical considerations surrounding the use of DNA as a vaccine need further consideration. Although it has not been an issue in clinical studies of naked DNA vaccines to date, the potential for a low frequency of integration of the vaccine DNA into the host chromosome or the induction of anti-DNA antibodies to cause disease remains a concern. Standard i.m. injection of naked DNA is very inefficient because only a small fraction of DNA is taken up by cells and expressed, but advances in delivery technologies such as ballistic delivery and electroporation are beginning to overcome this barrier. Indeed, our results suggest that vaccinating at different sites with multi-type L1 or L1+L2 DNA vaccines and using electroporation to enhance delivery shows promise as a next generation HPV vaccine candidate.
